# Association Between Vaginal *Gardnerella* and Tubal Pregnancy in Women With Symptomatic Early Pregnancies in China: A Nested Case-Control Study

**DOI:** 10.3389/fcimb.2021.761153

**Published:** 2022-01-17

**Authors:** Yingxuan Zhang, Si Chen, Xiaofeng Chen, Huimin Zhang, Xuge Huang, Xiaomeng Xue, Yinan Guo, Xiaofeng Ruan, Xiaorong Liu, Gaopi Deng, Songping Luo, Jie Gao

**Affiliations:** ^1^ The First Clinical College, Guangzhou University of Chinese Medicine, Guangzhou, China; ^2^ Lingnan Medical Research Center, Guangzhou University of Chinese Medicine, Guangzhou, China; ^3^ Department of Gynecology, The First Affiliated Hospital of Guangzhou University of Chinese, Guangzhou, China

**Keywords:** tubal pregnancy, vaginal microbiota, *Gardnerella*, symptomatic early pregnancy, China

## Abstract

The early diagnosis and treatment of ectopic pregnancy (EP) remains a major challenge. Despite a known link between vaginal microbiota and female reproductive health, few studies have focused on the association between vaginal microbiota and pregnancy location. This nested case-control study aimed to characterize the vaginal microbiota in tubal pregnancy (TP) among symptomatic women in early pregnancy. Women with symptomatic early pregnancy of unknown location (PUL) were included in this study. 16S rDNA gene sequencing was performed to assess vaginal microbial diversity and relative abundance. Machine learning and multivariate logistic regression were also used to evaluate the association between *Gardnerella* and TP. The results indicate that the vaginal microbiome in TP was more diverse (Shannon, *p* < 0.05) and was different in composition to that of women with intrauterine pregnancy (IUP) (weighted Unifrac, *R* = 0.08, *p* = 0.01). The genus *Gardnerella* was significantly enriched in TP. The XGBoost analysis was able to classify *Gardnerella*-induced TP more reliably (AUC = 0.621). Moreover, after adjusting potential confounders, our results indicate a robust association between *Gardnerella* and TP (as a continuous variable, adjusted OR: 12.0, 95% CI: 2.1–67.4, *p* < 0.01; as a categorical variable (≥0.85%), and adjusted OR: 4.2, 95% CI: 2.0–8.8, *p* < 0.01). In conclusion, we found that higher virginal *Gardnerella* levels were associated with TP in women with symptomatic early pregnancy.

## Introduction

Ectopic pregnancy (EP) is defined as pregnancy occurring outside the uterine cavity (ACOG Practice Bulletin No. 193: Tubal Ectopic Pregnancy, 2018). Over 95% of EP cases occur in the fallopian tube (Marion and Meeks, 2012), which is defined as tubal pregnancy (TP). The incidence of EP in the USA is 1%–2%; however, some studies indicate that this may be underestimated due to the difficulty of tracking patients with EP who have been treated in an outpatient setting (Hendriks et al., 2020, Ucisik-Keser et al., 2021). TP remains the most common cause of maternal morbidity and mortality, deeply affecting patients’ physical and mental state and resulting in approximately 2.7% of pregnancy-related deaths (Creanga et al., 2017). Therefore, early diagnosis and intervention are essential for safeguarding pregnant women against these complications. Although some progress has been made, there are currently no standard measures in place for the early diagnosis of tubal implantation (Carusi, 2019; Chen et al., 2020; Sahin et al., 2021). Although the mechanism of TP is complex, the majority of relevant studies indicate that inflammation of the fallopian tube or the surrounding pelvic tissues, due to factors such as the presence of pelvic inflammatory disease (PID) or *Chlamydia trachomatis* (CT) infection (Xia et al., 2020), is central to TP development (Shao et al., 2009; Al-Azemi et al., 2010; Li et al., 2013). Therefore, the discovery of inflammation-associated biomarkers could help predict TP development. Moreover, a nested case-control study with a large samples size (*N* = 2,026) has revealed that abdominal pain [odds ratio (OR): 1.16, 95% confidence interval (CI): 0.92–1.48] and bleeding (OR:1.34, 95% CI: 1.04–1.78) are risk factors for EP (Xia et al., 2020). The incidence of EP among pregnant women experiencing vaginal bleeding or abdominal pain (or both) in the first-trimester of pregnancy has been shown to be as high as 18% (Barnhart et al., 2006). Therefore, in the present study, we focused our attention on symptomatic pregnant women in the early stages of pregnancy.

The human commensal microbiome has evolved to coexist with the human genome and contributes to the maintenance of human health. Recently, the Human Microbiome Project (HMP) has shown that vaginal microbiota (VM) account for approximately 9% of the whole human microbiome (Huttenhower et al., 2012; Methé́ et al., 2012b; Gonzalez et al., 2014). While the most abundant vaginal bacteria are *Lactobacilli*, other genera, including *Gardnerella*, *Prevotella*, *Bifidobacterium*, *Atopobium*, *Megasphaera*, *Sneathia*, and *Anaerococcus* also play important roles as part of the vaginal microbiome (Huttenhower et al., 2012a; Methé́ et al., 2012b). 16S rRNA gene sequencing has been widely used to assess bacterial diversity and composition (Woo et al., 2008; Siddiqui et al., 2011; Jiao et al., 2021). This tool can help clinicians to find uncultivable bacteria and evaluate new pathogens that could serve as biomarkers in various disease settings (Yoshimura et al., 2011). As a result, large efforts have been made to assess the bacterial composition and diversity of the healthy versus abnormal vaginal microbiome (Franasiak and Scott, 2015; Moreno and Simon, 2019). In addition, vaginal microbial dysbiosis was shown to be associated with several female reproductive disorders, including premature delivery (Kumar et al., 2021), premature rupture of membranes (Amabebe et al., 2016), infertility (Sharma et al., 2014), PID (Rosca et al., 2020), *in vitro* fertilization (IVF) failure (Karaer et al., 2021), and recurrent miscarriage (Jiao et al., 2021). It is therefore of outmost importance to understand the vaginal microbiological characteristics of pregnant women, which are especially poorly described early on in pregnancy when fetal location is unknown, and represents a critical period for the early diagnosis of TP.

In this study, we aimed to assess the potential association between vaginal microbial composition and the incidence of TP among symptomatic pregnant women who were initially considered to have a pregnancy of unknown location (PUL), in China. In order to enhance the analytical power of our research, we employed both the 16S rRNA gene sequencing and machine learning methods.

## Materials and methods

### Ethical Approval

This study was approved by the Ethics Committee of the First Affiliated Hospital of Guangzhou University of Chinese Medicine (grant number ZYYECK2017-060), Guangzhou, China. All study participants gave their informed consent on meeting the inclusion criteria. All study procedures were in line with the Declaration of Helsinki.

### Study Design

This nested case-control study is a secondary analysis of a follow-up symptomatic cohort of pregnant women in Guangdong, China. Between May 2018 and December 2020, we enrolled pregnant women who were initially diagnosed as having PUL, at the First Affiliated Hospital of Guangzhou University of Chinese Medicine. The inclusion criteria were as follows: (1) diagnosed as PUL (positive pregnancy test but the pregnancy cannot be located by transvaginal ultrasonography) (Bobdiwala et al., 2019); (2) aged ≥18 years; (3) presented with abdominal pain or/and bleeding; and (4) gestational age of 4–8 weeks. The exclusion criteria were as follows: (1) patients had taken antibiotics within 30 days of vaginal sample collection; (2) patients had performed vaginal douching, sexual activity, or recorded use of vaginal drugs within 48 h of vaginal sample collection; and (3) patients had acute inflammation, cancer, or vulvovaginal candidiasis.

Participants in this study were followed up until their pregnancy location was determined. In line with existing clinical guidelines, the diagnosis of TP and intrauterine pregnancy (IUP) was based on symptoms, signs, previous medical history, laboratory examination, and transvaginal ultrasonography. The diagnostic criteria for IUP were confirmed by the presence of a yolk sac or embryo in the intrauterine gestational sac (Barnhart et al., 2011). The diagnosis of TP was validated by laparoscopy and histopathology (ACOG Practice Bulletin No. 193: Tubal Ectopic Pregnancy, 2018). In our study, the term “cases” defined patients with diagnosed TP, while “controls” referred to participants diagnosed with IUP in the same cohort. We matched cases and controls at a ratio of 1:2, while controlling for age (± 5 years) and gestational age (± 7 days) when collecting vaginal sample (Filion et al., 2016). We calculated that we would need to assign 31 patients to the TP group and 62 patients to the IUP group to enable the study to have 80% power to detect a minimally significant difference in vaginal microbiome composition between the two groups, at an alpha level of 0.05.

### Collection of Clinical Data and Vaginal Sampling

When first presenting at the outpatient clinic, participants were asked to complete a case report form including parameters such as baseline characteristics, previous reproductive history, previous medical history, lifestyle, and symptoms. A physical examination and collection of vaginal secretions were also performed. The process of vaginal sample collection was performed by an experienced gynecologist. Three sterile swabs (Improve Medical, Guangzhou, China) were used to collect each participant’s vaginal sample, so that results could be obtained in triplicate. A total of five samples were collected from various sides of the midvaginal canal. One swab was used to exclude vulvovaginal candidiasis by wet mount microscopy (Workowski and Bolan, 2015). A second swab was used to immediately measure the pH of the vaginal secretions. A further swab was frozen at −20°C within 4 h after sample collection, prior to being transferred for long-term storage at −80°C until DNA extraction.

### DNA Extraction and Processing of 16S rRNA Sequencing Data

Total genomic DNA was prepared using the DNeasy PowerSoil Kit (QIAGEN, Hilden, Germany). Agarose gel electrophoresis was used to verify the DNA concentration. The eligibility criteria for library construction and sequencing included: (1) the obvious presence of a main DNA band; (2) DNA concentration >10 ng/µl; and (3) optical density (OD) 260/280 >1. We used AMPure XP beads (Agencourt) for purification and performed another round of PCR amplification for qualified samples. After the second round of purification with AMPure XP magnetic beads, the final amplicon was quantified using the Qubit dsDNA detection kit. The sequencing step was initiated after merging equal amounts of purified amplicons. The amplified region is the corresponding region for bacterial diversity identification; the 16S V3–V4 region (upstream primer 343F: 5′TACGGRAGGCAGCAG-3′, downstream primer 798R: 5′-AGGGTATCTAATCCT3′). The samples were sequenced using the Illumina MiSeq platform, and the sequencing strategy was PE300.

Raw sequencing data were in the FASTQ file format. We used Trimmomatic (version 0.35) (Bolger et al., 2014) software to perform primer and label removal and quality control. FLASH (version 1.2.11) (Magoc and Salzberg, 2011) software was used to splice double-ended sequences with an overlap of 10–200 bp and a mismatch rate of <20% to form a complete paired-end sequence. The Split_libraries (version 1.8.0) (Caporaso et al., 2010) module in the QIIME software was used to remove ambiguous N bases and sequences with single base repeats >8 and a length of <200 bp, to obtain clean tags. After using UCHIME (version 2.4.2) (Edgar et al., 2011) to remove the chimera, the valid tags that were divided into operational taxonomic units (OTU) (Blaxter et al., 2005) are used in subsequent analysis. After the preprocessing was completed, Vsearch (version 2.3.2) (Blaxter et al., 2005) software was used to cluster the sequences with similarity ≥97% and merge them into one OTU. We selected the most enriched sequence as the representative sequence, then compared it against the SILVA and Greengenes database (DeSantis et al., 2006) for classification and annotation.

### Other Covariates Involved in This Study

The following variables were used: age, body mass index (BMI), smoking history, gestational age, symptoms (i.e., uterine bleeding and abdominal pain), menstrual cycle length, history of gestation (i.e., gravidity, previous pregnancy loss, and previous ectopic pregnancy), history of surgery (i.e., previous uterine cavity surgery and previous pelvic surgery), previous history of pelvic inflammatory disease (PID), and vaginal pH at baseline. We also collected demographic data, general information, variables that can affect vaginal microbiota, or risk factors of TP incidence previously reported in the literature (Bobdiwala et al., 2019; Hendriks et al., 2019; Hendriks et al., 2020; Mummert and Gnugnoli, 2021).

### Statistical Analysis

According to the number of sequences contained in the OTU for each sample, an OTU matrix file was created for subsequent analysis. Alpha diversity (using Shannon and Simpson Diversity Indices) was performed to evaluate the within-community diversity. We also used beta diversity (using weighted Unifrac and Bray Curtis tests) to analyze the variation in community composition (Lozupone et al., 2011). Both diversity values were calculated using QIIME. Principal component analysis (PCoA) was employed to further assess the differences in microbial community composition. We also performed linear discriminant analysis (LDA). Effect size (LEfSe) with default parameters (the alpha value for Wilcoxon tests was 0.05, the threshold on the logarithmic LDA score was 2.0) was implemented to find microbial species that were significantly different between the experimental groups (Segata et al., 2011).

To further evaluate the importance of different microbial species in TP, we undertook XGBoost, a machine learning method, using the relative abundance of genera. The accuracy of detecting different species was compared by the average area under the receiver operating characteristic (ROC) curve (AUC). Furthermore, multivariate logistic regression analysis was used to assess the association between the relative abundance of *Gardnerella* and TP. Nonadjusted, minimally adjusted, and fully adjusted models were listed. Covariances added in the adjusted model all had matching odds ratio changes ≥10%. Both the XGBoost analysis and multivariate logistic regression analysis were performed using EmpowerStats (www.empowerstats.com/).

Statistical analysis of cohort baseline characteristics and indices of alpha diversity was performed by SPSS 22.0 (IBM, Armonk, NY, USA). Differences between groups were calculated using Student’s *t*-test for continuous variables with a normal distribution, the Wilcoxon test for skewed continuous variables, and the Chi-square test of Fisher’s exact test for categorical variables. Values with a *p* < 0.05 (two-tailed) were considered significant.

## Results

### Study Population

**Figure 1:**
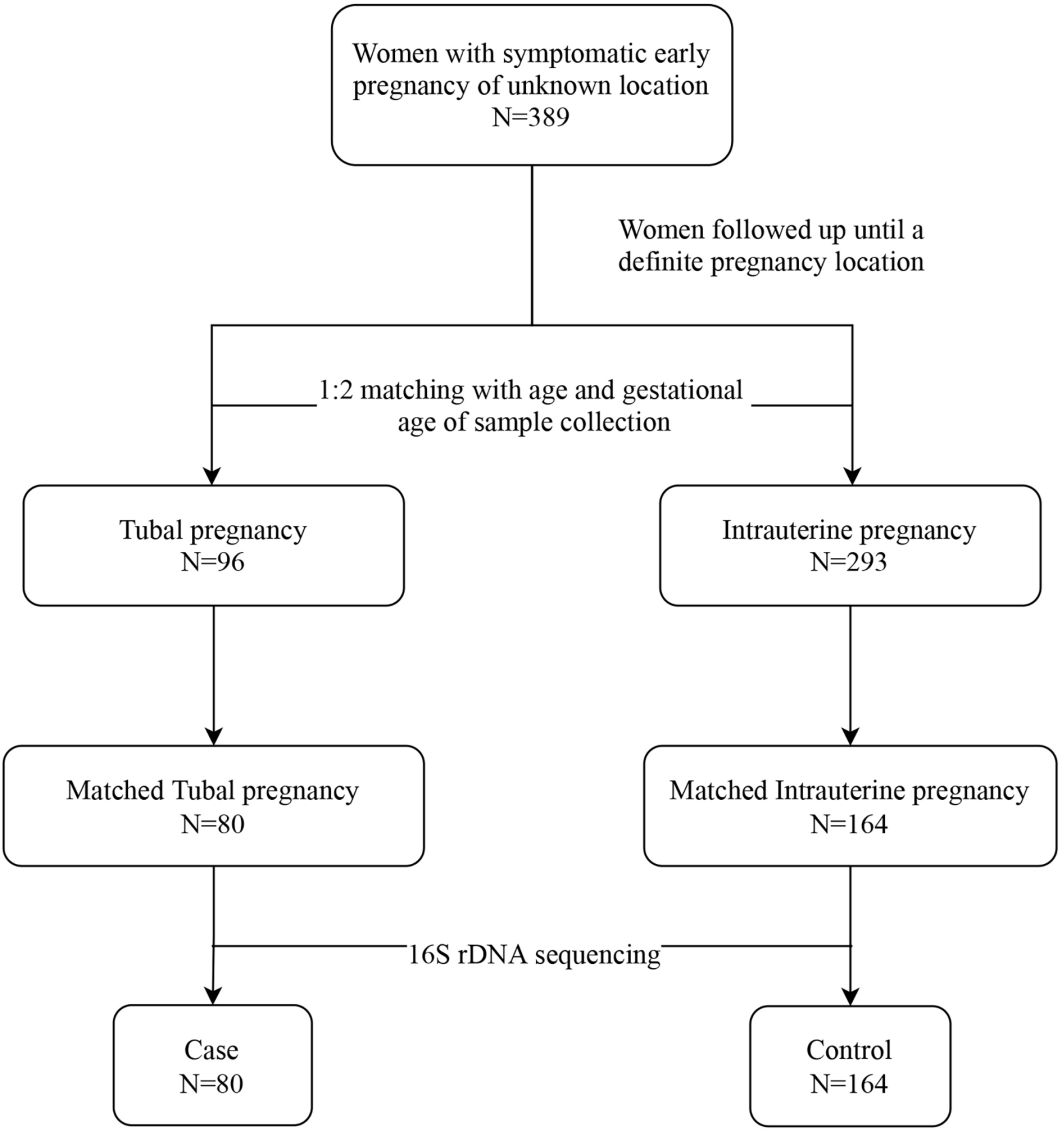
The study flowchart.

We assembled a baseline cohort of 389 women with symptomatic early PUL. Women whose pregnancy location had been identified were excluded from the study. Patients were followed from when they entered the cohort until they were diagnosed with IUP or TP. Ninety-six patients were confirmed as having TP, while 293 women were diagnosed with IUP. According to age and gestational age, 80 women who were diagnosed with TP (labeled “cases”) were matched with 164 women with IUP (labeled “controls”), at ratio of 1:2, and deemed eligible for 16S rRNA sequencing (**Figure 1**).

Among the study population, women who had confirmed TP were predominantly more likely to have symptoms of vaginal bleeding and abdominal pain than those in the IUP group. Women in the cases group also had a shorter menstrual cycle, more gravidity, previous ectopic pregnancies, and were more susceptible to PID. However, more of the women in the IUP group had a history of pelvic surgery. The two groups were otherwise similar in age, BMI, gestational age, history of pregnancy loss, and uterine cavity surgery (**Table 1**).

**Table 1:** Participant baseline characteristics.

	Intrauterine pregnancy	Tubal pregnancy	*p*-value
Number	164	80	
Age (years)	29.8 ± 5.5	30.4 ± 5.2	0.39
BMI (kg/m^2^)	28.0 ± 6.5	29.6 ± 5.9	0.07
Current or ex-smoker (yes)	0 (0.0%)	1 (1.2%)	0.15
Gestational age (days)	48.2 ± 7.9	46.4 ± 6.8	0.09
Uterine bleeding (yes)	64 (43.5%)	66 (82.5%)	<0.01^*^
Abdominal pain (yes)	47 (30.3%)	55 (68.8%)	<0.01^*^
Menstrual cycle (days)	37.2 ± 14.1	30.5 ± 5.8	0.01^*^
Gravidity	2.5 ± 1.4	3.2 ± 1.9	0.01^*^
Previous pregnancy loss (yes)	65 (39.9%)	34 (42.5%)	0.70
Previous ectopic pregnancy (yes)	4 (2.6%)	20 (25.3%)	<0.01^*^
Previous uterine cavity surgery (yes)	50 (52.1%)	24 (54.5%)	0.79
Previous pelvic surgery (yes)	28 (17.2%)	26 (32.5%)	0.01^*^
Previous pelvic inflammatory disease (yes)	23 (14.1%)	29 (36.7%)	<0.01^*^
Vaginal environment			
Vaginal pH	4.1 ± 1.5	4.4 ± 1.5	0.20
*Gardnerella* relative abundance	0.1 ± 0.2	0.1 ± 0.2	0.02^*^
*Gardnerella* relative abundance ≥0.0085 (yes)	31 (18.9%)	37 (46.2%)	<0.01^*^

Continuous variables were presented as mean ± SD; categorical variables were expressed as percentages (%).

p was calculated by t-test for normally distributed continuous variables, Chi-square test or Fisher’s exact test for categorical variables.

BMI, body mass index.

^*^p < 0.05.

### Characteristics of the Vaginal Microbiota of IUP and TP Women

**Figure 2:**
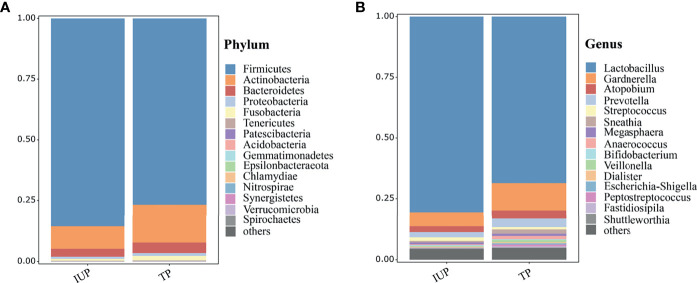
Vaginal taxonomic profiles of women with IUP or TP. **(A)** Vaginal microbiome profiles of women who were confirmed as having IUP or TP, at phylum level; the top 15 taxa are showed in the figure. **(B)** At the gene level, the top 15 taxa are listed according to their relative abundances in the two groups.

We obtained vaginal samples from 80 TP and 164 IUP women within the basal cohort. The vaginal microbial composition was similar in women with TP or IUP but differed in the relative abundances of bacterial species. Note, that for the purposes of clear visualization, we only presented the top 15 taxa (at the phylum and genus levels), and genera that fell outside these top 15 were merged together as and labelled “others” (**Figure 2**). As **Figure 2** shows, the vaginal microbiome of both the IUP and TP groups was dominated by *Firmicutes* (IUP 86% *vs*. TP 77%), *Actinobacteria* (IUP 10% *vs*. TP 15%), and *Bacteroidetes* (IUP 3% *vs*. TP 4%). At the genus level, the species composition of the IUP and TP groups were similar, but the relative abundance of each species differed. They were mainly composed of *Lactobacillus* (IUP 81% *vs*. TP 69%), *Gardnerella* (IUP 6% *vs*. TP 11%), *Atopobium* (IUP 2% *vs*. TP 3%), *Prevotella* (IUP 2% *vs*. TP 3%), *Streptococcus* (IUP 1% *vs*. TP 1%), and *Sneathia* (IUP 1% *vs*. TP 1%). We next conducted a diversity analysis. Shannon and Simpson indices were used for alpha diversity comparison. Women who were diagnosed with IUP had a higher Simpson index than the TP group (*p* < 0.05) but exhibited no significant difference in the Shannon index, meaning that the evenness of the vaginal microbiome was higher for the IUP group than the TP group, but the richness of two groups was similar (**Supplementary Figure S1**). The vaginal bacteria identified in the IUP and TP groups were separated into PCoA based on weighted Unifrac distances. ANOSIM showed that the two study groups were significantly different (*R* = 0.08, *p* = 0.01), indicating the heterogeneity of comparison between the two (**Supplementary Figure S2**).

**Figure 3:**
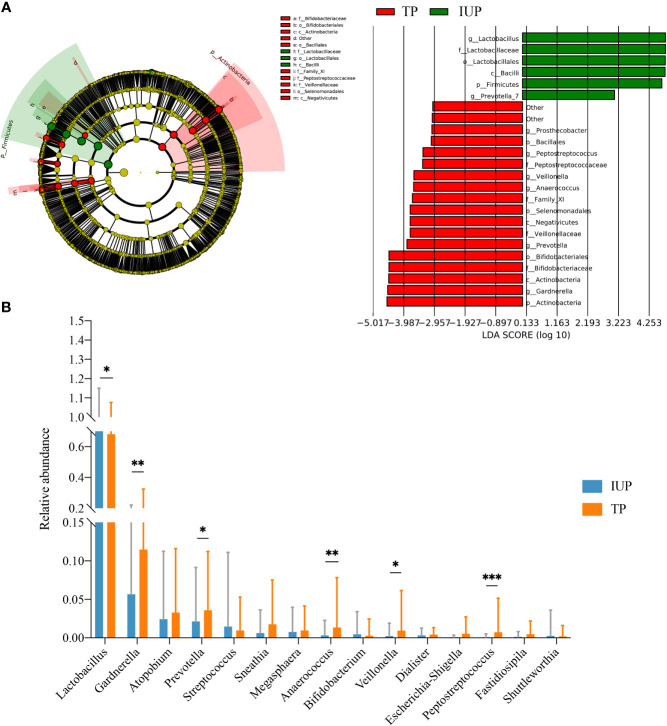
Differences in vaginal microbiota composition between IUP and TP women. **(A)** Differences detected in the vaginal microbiota of IUP and TP groups, using LEfSe analysis. Bacteria with LDA scores >2.0 are shown. **(B)** The relative abundance levels of *Lactobacillus*, *Gardnerella*, *Atopobium*, *Prevotella*, *Streptococcus*, *Sneathia*, *Megasphaera*, *Anaerococcus*, *Bifidobacterium*, *Veillonella*, *Dialister*, *Escherichia-Shigella*, *Peptostreptococcus*, *Fatidiosipila*, and *Shuttlemorthia* were compared in individuals with TP or IUP. The *p*-value was determined by two-tailed Wilcoxon’s rank-sum test. * represented P value <0.05, ** represented P value <0.01, *** represented P value <0.001.

To further explore the potential differences in the microbial features between women with IUP or TP, LEfSe analysis showed that six taxa were enriched in the IUP group. *Actinobacteria*, *Gardnerella*, *Bifldobacteriaceae*, and 13 other taxa were enriched in the TP group (**Figure 3A**). We next combined the results of the LEfSe analysis with relative abundance data. We selected the bacteria screened using LEfSe analysis and meet the top 15 relative abundances to compare the relative abundance of the IUP and TP groups. The relative abundance of *Lactobacillus* in the IUP group was significantly higher than in the TP group (*p* < 0.05). By contrast, the relative abundances of *Gardnerella*, *Prevotella*, *Anaerococcus*, *Veillonella*, and *Peptostretococcus* in the IUP group were lower than in the TP group (*p* < 0.05) (**Figure 3B**). These findings corroborate the notion that these types of bacteria may be useful biomarkers for distinguishing between IUP and TP in early pregnancy.

### *Gardnerella, Lactobacillus*, and *Aerococcus* Are Potential Biomarkers for Predicting Pregnancy Location

**Figure 4:**
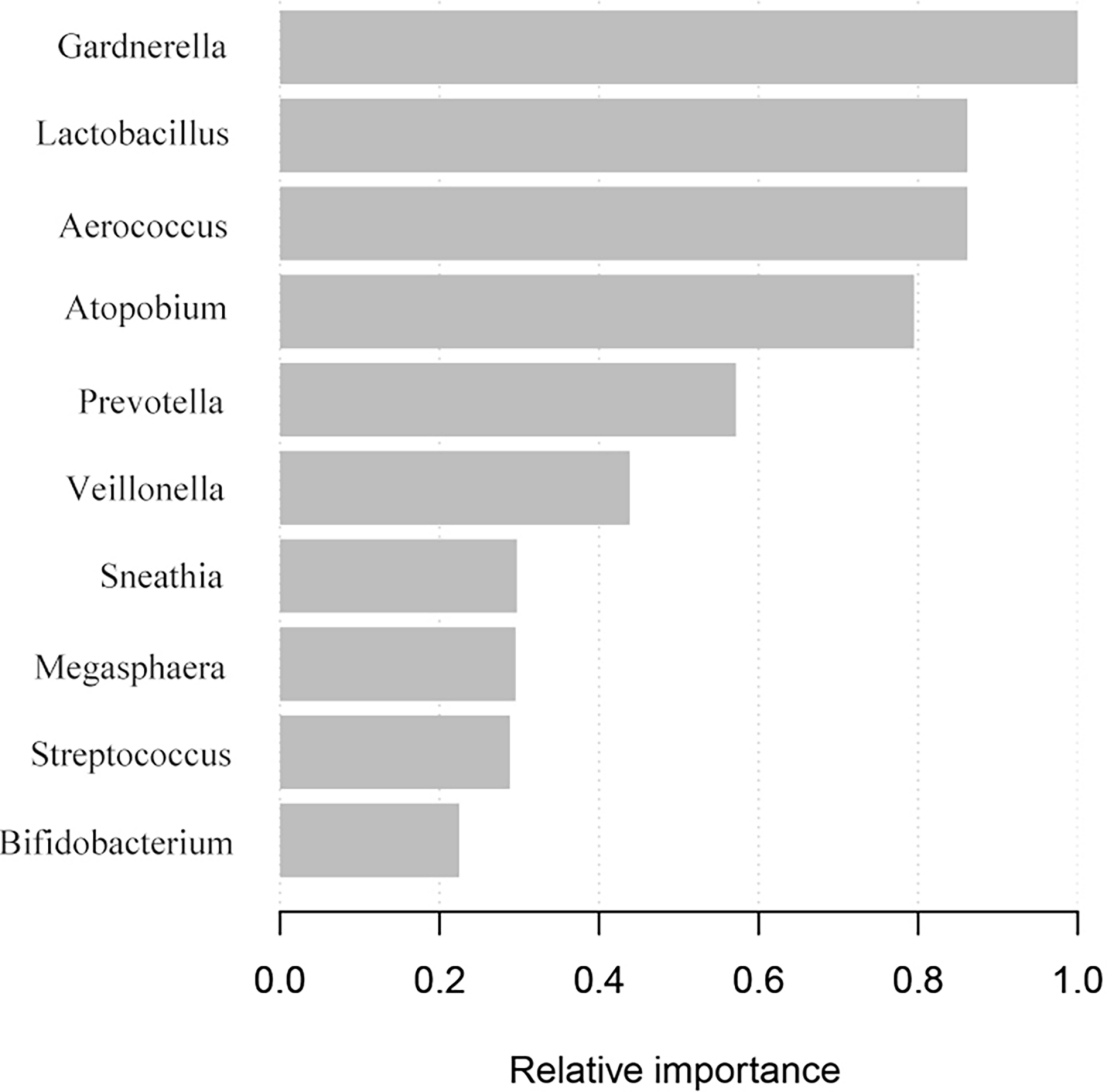
Screening of vaginal microbiota that contributed to TP diagnosis. The XGBoost machine learning tool utilized vaginal microbiome data to diagnose early pregnancy location.

**Figure 5:**
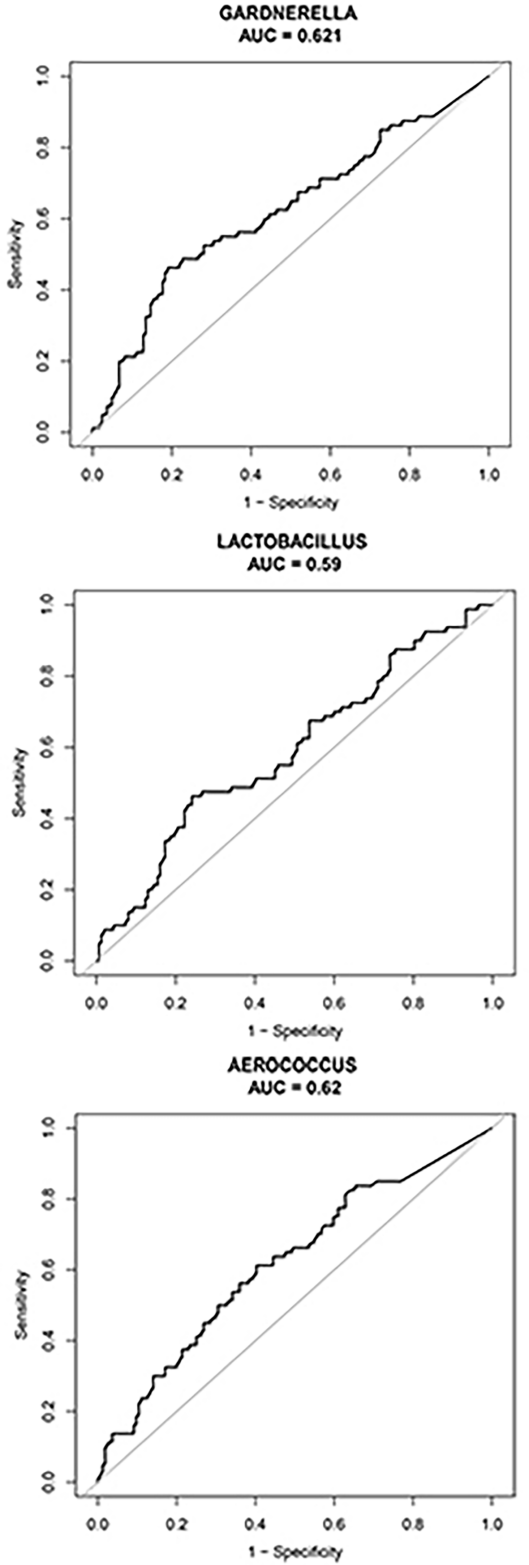
ROC was used to assess the diagnostic value of the *Gardnerella*, *Lactobacillus*, and *Aerococcus* genera. The black curve indicates average AUC for the three bacterial genera indicated. The diagonal lines mark an area under the receiver operating characteristic curve of 0.5.

We compared vaginal taxonomic profiles of the IUP and TP groups. The results indicated that bacteria such as *Gardnerella*, *Prevotella*, *Anaerococcus*, and *Veillonella* and further clarify the value of these bacteria in the diagnosis of early pregnancy location and the degree of contribution. We further employed different bacteria between the two groups by using XGBoost. *Gardnerella*, *Lactobacillus*, and *Aerococcus* (listed in order of importance) emerged as potential biomarkers for predicting the location of early pregnancy (**Figure 4**). The microbiota-based models also helped us to distinguish between individuals with IUP or TP. ROC was used to assess the diagnostic values for the three key bacterial subsets. The average AUC for *Gardnerella*, *Lactobacillus*, and *Aerococcus* was 0.621, 0.59, and 0.62, respectively (**Figure 5**). Combined with results of LEfSe analysis, we found that the relative abundance of *Gardnerella* had a strong correlation with TP, with a best threshold of 0.85% (**Supplementary Table S1**).

### The Relative Abundance of Vaginal *Gardnerella* Was Strongly Associated With TP

Multiple regression analysis was used to estimate the association between the relative abundance of *Gardnerella* and TP. The crude model showed that the relative abundance of *Gardnerella* was positively associated with the risk of TP [OR, 4.9; 95% CI, 1.2–20.5; *p* = 0.028]. The “adjusted I model”, adjusted for BMI only, presented the same trend as the crude model [OR, 5.7; 95% CI, 1.4–24.1; *p* = 0.018]. The “adjusted II model”, which was adjusted for BMI, abdominal pain, previous ectopic pregnancy history, and vaginal pH, also showed that the relative abundance of *Gardnerella* was strongly correlated with the risk of TP [OR, 12.0; 95% CI, 2.1–67.1; *p* = 0.005]. For the sensitivity analysis, we further used 0.85% as the cut point of the relative abundance of *Gardnerella*. The results of our crude model showed that women whose relative abundance of vaginal *Gardnerella* was >0.85% had a 2.7 times higher risk of developing TP than those with a value of <0.85% [OR, 4.2; 95% CI, 2.0–8.8; *p* < 0.001]. The same trend was confirmed in the adjusted I and II models (**Table 2**).

**Table 2:** Multivariate logistic regression analysis for the relationship between the relative abundance of *Gardnerella* and TP.

	Crude model (OR (95% CI), *p*)	Adjusted I model (OR (95% CI), *p*)	Adjusted II model (OR (95% CI), *p*)
*Gardnerella*	4.9 (1.2, 20.5), 0.028	5.7 (1.4, 24.1), 0.018	12.0 (2.1, 67.4), 0.005
*Gardnerella* group			
<0.85%	1.0	1.0	1.0
≥0.85%	3.7 (2.1, 6.6), <0.001	3.7 (2.1, 6.7) < 0.001	4.2 (2.0, 8.8) < 0.001

Crude model: not adjusted.

Adjusted I model: adjusted for BMI.

Adjusted II model: adjusted for: BMI, abdominal pain, previous ectopic pregnancy, and vaginal pH.

BMI, body mass index.

## Discussion

TP is still the major cause of hemorrhage-related pregnancy-associated death to date (Segata et al., 2011), and a recent study revealed that the global incidence of TP was on the rise (Goller et al., 2018). The underlying risk factors associated with TP are complex, including previous incidences of PID, EP, tubal surgery, infertility, and *Chlamydia trachomatis* infection (ACOG Practice Bulletin No. 193: Tubal Ectopic Pregnancy, 2018; Marion and Meeks, 2012; Rana et al., 2013). Numerous studies have indicated that chronic inflammation in the tissues surrounding the female reproductive tract plays a crucial role in the incidence of TP (Marion and Meeks, 2012; Rana et al., 2013; ACOG Practice Bulletin No. 193: Tubal Ectopic Pregnancy, 2018). However, 50% of patients with TP have no definitive risk factors (Ankum et al., 1996; Barnhart et al., 2006; ACOG Practice Bulletin No. 193: Tubal Ectopic Pregnancy, 2018), a statistic which continues to confound gynecologists.

Recently, an increasing number of studies have found that microbes play an important role in human physiology and pathology alike (Filardo et al., 2017; Di Pietro et al., 2018). As an important component of the human microbiome, vaginal microbiota play a critical role in female reproduction and fertility (Franasiak and Scott, 2015; Moreno and Simon, 2019). The development of advanced sequencing analysis has enabled us to expand our knowledge of vaginal microbial composition in order to delineate the relationship between microbial perturbations in pregnancy and adverse pregnancy outcomes. However, few studies have focused on examining the characteristics of the vaginal microbiome in patients with TP. Further characterizing the vaginal microbiome may help gynecologists to identify which women in the PUL period have a higher risk of developing TP. Several studies have shown that vaginal microbiota became less diverse, more stable, and were dominated by *Lactobacillus* in women with normal pregnancies (DiGiulio et al., 2015). Consistent with the results of our studies, some findings have also indicated that pregnancy complications were associated with the increased relative abundance of anaerobes, including *Gardnerella*, *Prevotella*, and *Atopobium* (Fettweis et al., 2019; Siena et al., 2021). Moreover, the most frequently described harmful change affecting the vaginal microbiome in women of reproductive age, bacterial vaginosis (BV), is characterized by a decrease in relative *Lactobacillus* abundance and an increase in anaerobic bacteria, primarily *Gardnerella vaginalis* (Fredricks et al., 2005). The presence of BV in early pregnancy was confirmed to be related to several adverse pregnancy outcomes, including preterm delivery (Petricevic et al., 2014), premature rupture of membranes (Purwar et al., 2001), recurrent miscarriage (Jiao et al., 2021), spontaneous abortion (Isik et al., 2016), and PID (Bagnall and Rizzolo, 2017). Although, the potential link between *Gardnerella* abundance and the incidence of TP has not been fully elucidated, we propose the following possible mechanism.

*Gardnerella vaginalis* infection is a large contributor of BV (Cheah et al., 2020), particularly in women of reproductive age (Wong et al., 2018). Approximately 40% of women with BV have no obvious symptoms and remain untreated (Klebanoff et al., 2004). Therefore, the harmful effects of *Gardnerella* infection are chronic and easily overlooked. It has also been reported that *Gardnerella* itself is associated with the development of several genital tract conditions, including endometriosis (Khan et al., 2016) and PID (Brunham et al., 2015). These findings support the hypothesis that *Gardnerella* may ascend into the upper reproductive tract and, thus, affect the incidence of TP. In fact, some studies have also supported this potential underlying mechanism. In women with normal pregnancies, hormonal alterations favor *Lactobacillus* proliferation, leading to increased level of antibacterial substances (e.g., H_2_O_2_ and bacteriocins) and lactic acid, which maintains the vaginal pH at 3.5–4.5 (Boskey et al., 1999; Greenbaum et al., 2019). However, when the relative abundance of *Gardnerella* increases, the neuraminidase enzymes generated by *Gardnerella* spp. can damage the membrane of cervical epithelial cells and weaken the barrier between the vagina and cervix, thus, raising the risk of cervical infections (Haahr et al., 2016). Nicole et al. reported that *Gardnerella vaginalis* stimulated group B *Streptococcus* (GBS) vaginal colonization, leading to ascending uteroplacental infection in pregnant mice (Gilbert et al., 2021). In addition, *Gardnerella* was shown to activate the local proinflammatory cytokine response and the innate immune system and lead to cervical remodeling (Sierra et al., 2018; Zheng et al., 2019; Florova et al., 2021). Fan and colleagues found that pathogenic bacteria, including *Gardnerella*, could trigger an imbalance in immune tolerance at the maternal-fetal interface through the perturbation of regulatory chemokine networks (Fan et al., 2020).

Our study revealed the potential association between vaginal microbiota and pregnancy location. However, translating these complex ecological metrics into the clinical setting is a challenge. Classification of vaginal microbiota effectively simplifies the complex biological dataset and may be helpful in epidemiological investigations and in disease diagnosis (Kindinger et al., 2016; Ravel et al., 2021). Several studies have indicated that the vaginal microbiota can be clustered by community state type (CST), which was developed by characterizing the diversity of bacterial taxa in the vaginal microbiome of ~400 multiethnic women of reproductive age (Ravel et al., 2011; Gajer et al., 2012). Four of the CSTs are dominated by *Lactobacillus*, including *Lactobacillus crispatus* (CST I), *Lactobacillus gasseri* (CST II), *Lactobacillus iners* (CST III), and *Lactobacillus jenseni*i (CST V) (Ravel et al., 2011; Drell et al., 2013). Among these, only CST IV comprises many subsets of anaerobic bacteria, including *Gardnerella*, *Atopobium*, *Prevotella*, *Mobiluncus*, and *Sneathia*. Therefore, CSTs have their limitations, one of which is that most of the CSTs are built around the presence of four definitive dominant *Lactobacillus* species. Crucially, approximately 25% of the women sampled clustered into CST IV (Ravel et al., 2011; Drell et al., 2013), therefore masking the detrimental effect of *Gardnerella*. Classification according to the relative abundances at the genus level could classify samples more definitively and may more effectively reveal associations between the vaginal microbiome and pregnancy outcome (Al-Memar et al., 2020). Robinson et al. pointed out that instead of clustering by classification, machine-learning algorithms could overcome such disadvantages (Robinson et al., 2016). Machine learning could effectively improve bioinformatics analysis for making the microbial-community groups independent of samples and supporting comparability between studies (LeCun et al., 2015). Nevertheless, few studies have applied machine learning to analyze vaginal microbiota. Extreme Gradient Boosting (XGBoost) is an efficient algorithm used for machine learning (Taylor et al., 2018; Ye et al., 2018) that has been widely applied to predict the onset of disease and even for providing genetic counseling for individuals. The predictive testing process employed by the XGBoost model is beneficial for evaluating the expecting onset, which is helpful for optimizing future medical plans (Peng et al., 2021). Therefore, we integrated LEfSe analysis and the XGBoost algorithm to efficiently process large-scale microbiological data, with the aim of predicting TP.

Few studies have compared the vaginal microbiota between IUP and TP groups. The population we included consisted of symptomatic pregnant women with PUL, who were at high-risk for TP and required special clinical attention. Our study revealed a significant difference in the relative abundance of *Gardnerella* in the vaginal microbiome of women with TP and IUP, identifying *Gardnerella* as a potential biomarker for predicting the incidence of TP in early pregnancy. Moreover, we generated a model to reliably predict the incidence of TP in women with PUL based on 16S rDNA gene sequencing and machine learning. Nevertheless, our study still has the following limitations. Firstly, we included a well-defined study population, so the results would be limited to pregnant women with abdominal pain and/or uterine bleeding. Secondly, because the composition of vaginal microbiota can be affected by ethnicity (Fettweis et al., 2019; Dunlop et al., 2021), further studies should be performed to verify whether the results of our study are applicable to women with symptomatic early pregnancies outside of China. Thirdly, our study involved association analysis research and focused specifically on describing the association between vaginal microbiota and the incidence of TP. Although we found that the relative abundance of *Gardnerella* could be a potential and valuable biomarker to identify TP, the underlying mechanism needs to be further explored.

## Conclusion

Compared with the IUP group, the vaginal microbiome of TP patients exhibited higher microbial diversity and a higher relative abundance of *Gardnerella*, which could represent a potential diagnostic biomarker for TP. Our results provide new insights into the intricate relationship between the vaginal microbiome in early pregnancy and incidence of TP.

## Data Availability Statement

The datasets presented in this study can be found in online repositories. The names of the repository/repositories and accession number(s) can be found below: NCBI SRA BioProject, Accession No. PRJNA737055.

## Ethics Statement

The studies involving human participants were reviewed and approved by the Ethics Committee of the First Affiliated Hospital of Guangzhou University of Chinese Medicine (No. ZYYECK2017-060). The patients/participants provided their written informed consent to participate in this study. Written informed consent was obtained from the individual(s) for the publication of any potentially identifiable images or data included in this article.

## Author Contributions

JG, GD, and SL designed and founded the study. YG, XC, XH, XX, YG, XR and XL recruited participants. SC, YG, XC, XH, XX and YG collected clinical data and samples as well as analyzed and interpreted the data. SC and YZ generated the figures and tables. SC wrote the first draft, which was further developed by YZ. All authors have read the final manuscript and have agreed to be held accountable for its content.

## Funding

The study was funded by the National Natural Science Foundation of China (grant number 81774358), the Guangzhou University of Chinese Medicine (grant numbers XK2019016 and 2019IIT33), the National administration of Traditional Chinese Medicine (National Chinese Medicine People’s Education Development (2018) No. 12), and Department of Finance of Guangdong province (grant number 2020B1111100003).

## Conflict of Interest

The authors declare that the research was conducted in the absence of any commercial or financial relationships that could be construed as a potential conflict of interest.

## Publisher’s Note

All claims expressed in this article are solely those of the authors and do not necessarily represent those of their affiliated organizations, or those of the publisher, the editors and the reviewers. Any product that may be evaluated in this article, or claim that may be made by its manufacturer, is not guaranteed or endorsed by the publisher.

## Supplementary Material

Supplementary Figure S1 Alpha diversity comparison of the TP and IUP groups. **(A)** Alpha diversity measures of the Simpson index, which responds to richness of the samples. **(B)** Shannon Index was used to compare alpha diversity for evenness of these samples.

Supplementary Figure S2 PCoA analysis of vaginal microbiota between IUP and TP women. **(A)** PCoA analysis based on Bray Curtis distance of the IUP and TP group data. **(B)** PCoA analysis based on weighted Unifrac distance of the IUP and TP group data.
