# Supplementary figures and images for: Association Between Vaginal Gardnerella and Tubal Pregnancy in Women With Symptomatic Early Pregnancies in China: A Nested Case-Control Study
Source: Front Cell Infect Microbiol. 2022 Jan 17;11:761153. doi: 10.3389/fcimb.2021.761153 (PMC8801712; doi:10.3389/fcimb.2021.761153)

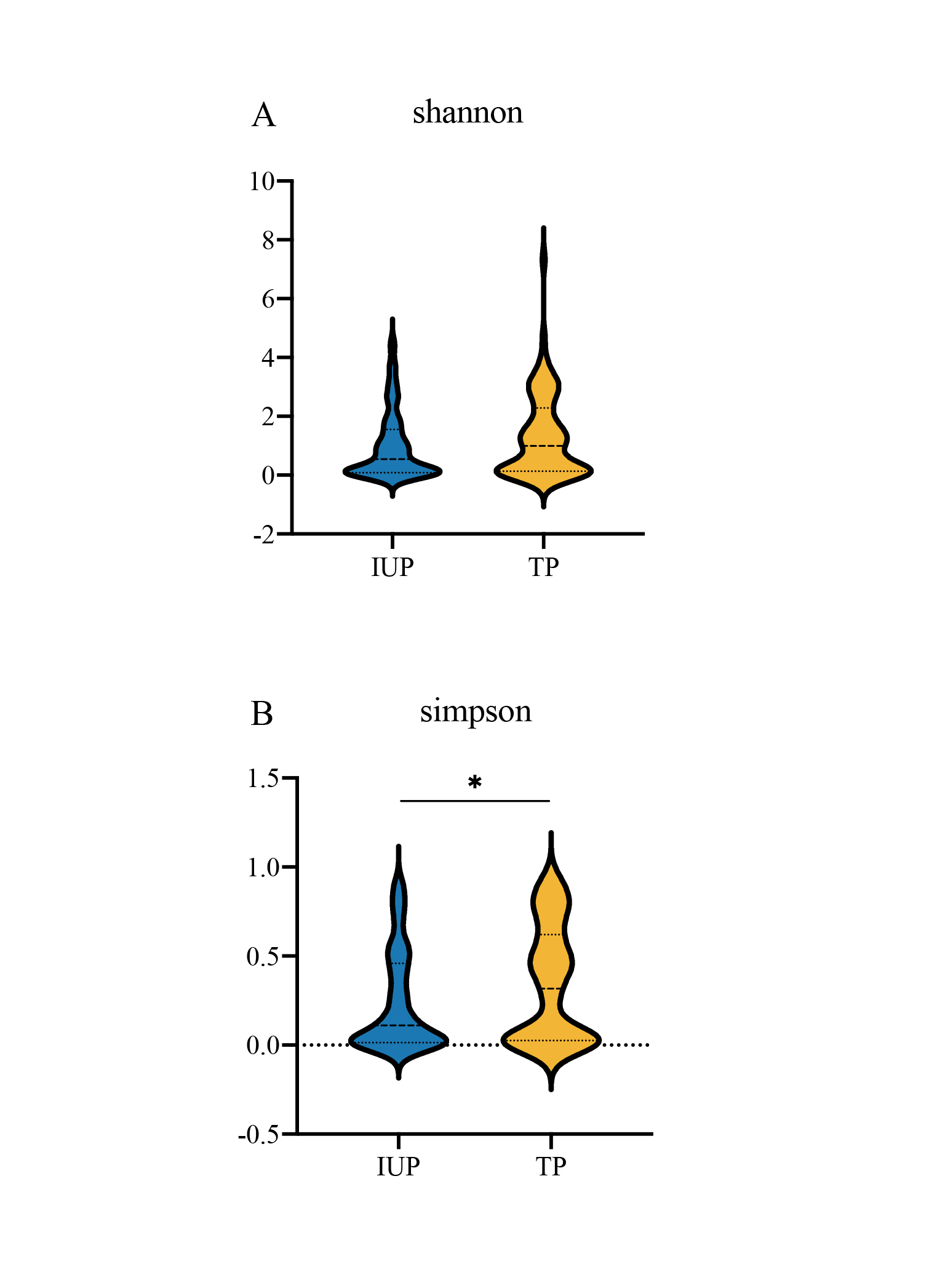

Supplement: Supplementary Figure S1 — Alpha diversity comparison of the TP and IUP groups. (A) Alpha diversity measures of the Simpson index, which responds to richness of the samples. (B) Shannon Index was used to compare alpha diversity for evenness of these samples. [file Image_1.jpeg]

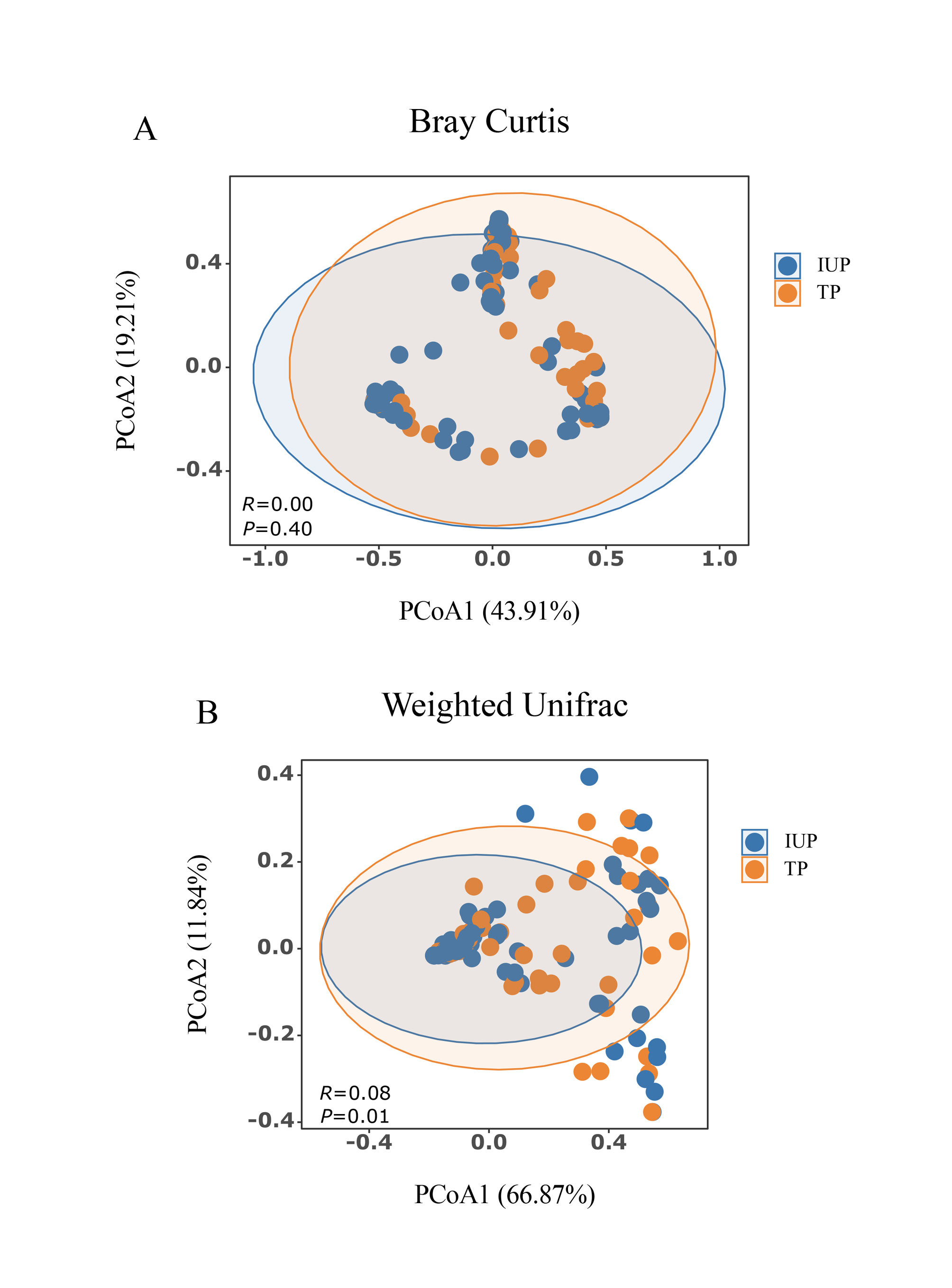

Supplement: Supplementary Figure S2 — PCoA analysis of vaginal microbiota between IUP and TP women. (A) PCoA analysis based on Bray Curtis distance of the IUP and TP group data. (B) PCoA analysis based on weighted Unifrac distance of the IUP and TP group data. [file Image_2.jpeg]
