# Supplementary material for: Association Between Vaginal Gardnerella and Tubal Pregnancy in Women With Symptomatic Early Pregnancies in China: A Nested Case-Control Study
Source: Front Cell Infect Microbiol. 2022 Jan 17;11:761153. doi: 10.3389/fcimb.2021.761153 (PMC8801712; doi:10.3389/fcimb.2021.761153)
Supplement: Supplementary file 3 [file Table_1.docx]

**Supplementary table I** Cut point of relative abundance of *Gardnerella* calculated by using xgboost

| **Test** | **TP** | **IUP** | **ROC area(AUC)** | **95%CI low** | **95%CI up** | **Best threshold** | **Specificity** | **Sensitivity** |
| --- | --- | --- | --- | --- | --- | --- | --- | --- |
| *Gardnerella* | 80 | 164 | 0.6210 | 0.5431 | 0.6988 | 0.0085 | 0.8110 | 0.4625 |
